# Induction of Paclitaxel Resistance by ERα Mediated Prohibitin Mitochondrial-Nuclear Shuttling

**DOI:** 10.1371/journal.pone.0083519

**Published:** 2013-12-23

**Authors:** Pei Dong, Lijuan Jiang, Jianye Liu, Zhiming Wu, Shengjie Guo, Ziling Zhang, Fangjian Zhou, Zhuowei Liu

**Affiliations:** 1 State Key Laboratory of Oncology in South China, Department of Urology, Sun Yat-sen University Cancer Center, Collaborative Innovation Center for Cancer Medicine, Guangzhou, Guangdong, China; 2 Guangdong Key Laboratory of Urology, The First Affiliated Hospital of Guangzhou Medical University, Guangzhou, Guangdong, China; University of Kentucky College of Medicine, United States of America

## Abstract

Paclitaxel is a drug within one of the most promising classes of anticancer agents. Unfortunately, clinical success of this drug has been limited by the insurgence of cellular resistance. To address this, Paclitaxel resistance was modeled in an *in vitro* system using estrogen treated prostate cancer cells. This study demonstrates that emerging resistance to clinically relevant doses of Paclitaxel is associated with 17-β-estradiol (E2) treatment in PC-3 cells, but not in LNCaP cells. We found that small interfering RNA mediated knockdown of ERα lead to a decrease in E2 induced Paclitaxel resistance in androgen-independent cells. We also showed that ERα mediated the effects of estrogen, thereby suppressing androgen-independent cell proliferation and mediating Paclitaxel resistance. Furthermore, E2 promoted Prohibitin (PHB) mitochondrial-nucleus translocation via directly mediation of ERα, leading to an inhibition of cellular proliferation by PHB. Additionally, restoration of Paclitaxel sensitivity by ERα knockdown could be overcome by PHB overexpression and, conversely, PHB knockdown decreased E2 induced Paclitaxel resistance. These findings demonstrate that PHB lies downstream of ERα and mediates estrogen-dependent Paclitaxel resistance signaling cascades.

## Introduction

Prostate cancer is one of the leading causes of death among men in developed countries. The primary treatment for hormone-refractory prostate cancer is taxane-based chemotherapy, including Paclitaxel [1]. Paclitaxel functions by stabilizing microtubule assembly and inhibiting depolymerization, thus causing mitotic arrest or aberrant mitosis. Higher concentrations of Paclitaxel can induce mitotic phase cell death, thereby exerting antitumor effects [2]. Taxane-based therapy often improves patient survival, however, the cancer ultimately develops drug resistance in most patients, leading to recurrence of the cancer, distant metastasis and death [3].

Several pathways are involved in progression to androgen independence in cases of advanced prostate cancer treated with hormone deprivation [4], increasing evidence that estrogen signaling has a major role in prostate cancer development and progression, often associated with estrogen receptor (ER) signaling [5], [6], [7], [8], [9]. Genomic modifications of the ER gene have been described, including amplification [8], [10] and mutation [11]. High-grade, primary Gleason grade 4 and 5 tumors revealed ER protein expression in 43% and 62% of cases, respectively [8]. Significant ERα gene expression as measured by mRNA and protein levels was observed in hormone refractory tumors and metastatic lesions, including lymph node and bone metastases [8]. These studies suggest that estrogen can affect prostatic cancerogenesis and neoplastic progression through an ER-mediated process in human prostate tissue. However, the mechanisms underlying estrogen and estrogen receptor signaling in human prostate tissue remain poorly understood.

PHB is ubiquitously expressed in all tissues tested to date and has been shown to have significant effects on cell senescence, cell development and tumor cell suppression [12], [13]. Data suggests that PHB can modulate the Rb–E2F transcription complex to repress E2F-mediated transcription and cell proliferation [14]. A significant correlation was found between low tumor cell proliferation and drug resistance. In non-Hodgkin's lymphomas, patients with tumor proliferation of less than 80% were significantly more likely than patients with rates of higher proliferation to be unresponsive to therapy or to fail to achieve a complete response, and tended to have a shorter period free of progression and lower overall survival [15]. Recently, Gregory-Bass *et al*. showed that repression of PHB in ovarian cancer cells increased their sensitivity to staurosporine [16]. Patel N *et al*. showed that stable and transient knockdown of PHB in a Paclitaxel-resistant lung cancer cell line or an uterine sarcoma cell line significantly improved sensitivity to Paclitaxel as well as to other chemotherapeutic agents in *vitro* and in *vivo* [17]. However, the mechanism underlying this PHB mediated Paclitaxel resistance remains unclear.

Our current work suggests that PHB is a mediator of E2-ERα induced Paclitaxel resistance. This resistance depends on the cellular localization of PHB, rather than on the absolute amount of the protein within the cell. These observations lead to the hypothesis that estrogen and PHB play a role in the development of drug resistance in prostate cancer.

## Materials and Methods

### Cell culture and treatment

LNCaP human prostate cancer cells were obtained from the American Type Culture Collection (Manassas, VA, USA) and cultured at 37°C in 5% CO_2_ in DMEM-F12 (1:1) media (Invitrogen) supplemented with 1% penicillin (100 U/ml, Invitrogen), 1% streptomycin (100 µg/ml, Invitrogen), L-glutamine (292 µg/ml, Invitrogen) and 5% fetal bovine serum (FBS; HyClone Laboratories). PC3 human prostate cancer cells were obtained from the American Type Culture Collection (Manassas, VA, USA) and were cultured as previously described [18]. Briefly, they were cultured in 5% CO_2_ at 37°C in RPMI 1640 (Invitrogen) supplemented with 1% penicillin (100 U/ml, Invitrogen), 1% streptomycin (100 µg/ml, Invitrogen) and 10% fetal bovine serum (FBS; HyClone Laboratories). 17-β-estradiol (E2, Cayman Chemical) and Paclitaxel (Abcam) were added to the media at the indicated concentrations for 96h and 24h, respectively. For transient gene transfection of cells, Lipofectamine 2000 was used.

### Cell viability analysis

Cells were stained with Hoechst 33258 (5 µg/ml) to visualize nuclei and propidium iodide (PI) (0.2 µg/ml) to detect membrane damage. Cell death was quantified by scoring the number of PI positive cells relative to the total number of nuclei within the same visual field. Cells, 1000 cells per group at minimum, were counted in an unbiased manner and were scored blindly without knowledge of which treatment they had undergone.

### RNA purification and RT-PCR analysis

Total RNA was extracted and isolated from cultured cells using TRIzol reagent (Invitrogen). Reverse transcription (RT) was performed using SuperscriptIII reverse transcriptase (Invitrogen) and oligo-dT primers. The following primer pairs were used: *erα*-F: 5`-TAC TGC ATC AGA TCC AAG GG-3` with *erα*-R: 5`-GTG GGA ATG ATG AAA GGT GG-3`, and *erβ*-F: 5`-TGA AAA GGA AGG TTA GTG GGA ACC-3` with *erβ*-R: 5`-TGG TCA GGG ACA TCA TCA TGG-3`.

### Immunoblotting

Cells were harvested at 4°C in Laemmli lysis buffer. After determining the protein content of the cell lysates, the protein extracts were separated by 10% SDS-PAGE, transferred to a PVDF membrane and incubated with primary antibody (ERα, ERβ, PHB and VDAC antibodies were from Santa Cruz Biotech, and Tubulin and Histone H1 antibodies were from Abcam). The signal was detected by ECL detection system (GE Healthcare).

### siRNA knockdown

siRNAs were used corresponding to the Human ERα, ERβ and PHB genes (Santa Cruz Biotech): sierα (Cat#: sc-29305), sierβ (Cat#: sc-35325), siphb (Cat#: sc-37629). siRNAs were transfected into cells using Lipofectamine 2000.

### DNA Growth Assay

Following treatment of cells, the media was discarded, cells were solubilized for 30 min at 37°C in 0.1% SDS and the amount of DNA was estimated using a Hoechst 33258 microassay, as extensively described previously [19].

### Subcellular fractionation

Approximately 10^7^ cells were harvested into 10 ml of isotonic fractionation buffer (250 mM sucrose, 0.5 mM EDTA, 20 mM Hepes, and 500 µM Na_3_VO_4_ at pH 7.2) supplemented with protease inhibitor cocktail complete (Roche Molecular Biochemicals) and centrifuged at 900 g for 5 min. The pellet was then resuspended in 200 µl fractionation buffer, homogenized with a ball-bearing homogenizer and centrifuged at 900 g for 5 min to remove the nuclei. The post-nuclear supernatant was centrifuged at 20,000 g for 15 min to collect the heavy membrane fraction enriched in mitochondria.

### Co-Immunoprecipitation

Cell extracts were prepared by solubilizing 10^7^ cells in 1 ml of cell lysis buffer made of 1% Triton X-100, 150 mM NaCl, 20 mM Tris-Cl at pH 7.4, 1 mM EDTA, 1 mM EGTA, 1 mM Na3VO4, 2.5 mM pyrophosphate, 1 mM glycerol phosphate and protease inhibitor mixture for 10 min at 4s°C. After brief sonication, the lysates were cleared by centrifugation at 15,000 g for 10 min at 4°C, the cell extract was immunoprecipitated with 6 µg of antibodies against ERα or PHB (antibodies were from Santa Cruz Biotech), and incubated with 100 µl of protein G plus protein A-agarose for 12h at 4°C by continuous inversion. Immunocomplexes were pelleted, washed 4 times, boiled in Laemmli buffer and analyzed by Western blot.

### Constructs

The plasmids pCDNA3.1-Prohibitin and pCDNA3.1-ERα were made by inserting Human Prohibitin or ERα cDNA into a pCDNA3.1 expression vector. Constructs were transfected into cells using Lipofectamine 2000.

### Statistical analysis

The statistics in the graphs represent the means with ± S.E. bars of at least three independent experiments. Each group was compared to the control using Student's t test. The significance is indicated as follows: * denotes p<0.05, ** denotes p<0.01, and *** denotes p<0.001.

## Results

### Estrogen inhibits Paclitaxel induced androgen-independent prostate cancer cell death

Human prostate cancer is considered a paradigm of an androgen-dependent tumor. However, the role of estrogen in malignant prostate cancer appears to be equally important. In animal model systems, estrogens, combined with androgens, appear to be required for the malignant transformation of prostate epithelial cells [20]. Although the mechanisms underlying the hormonal induction of prostate cancer *in vivo* remain uncertain, there is evidence to support that long term administration of androgens and estrogens results in an estrogenic environment in rat prostates and the ensuing development of cancer [20].

**Figure 1:**
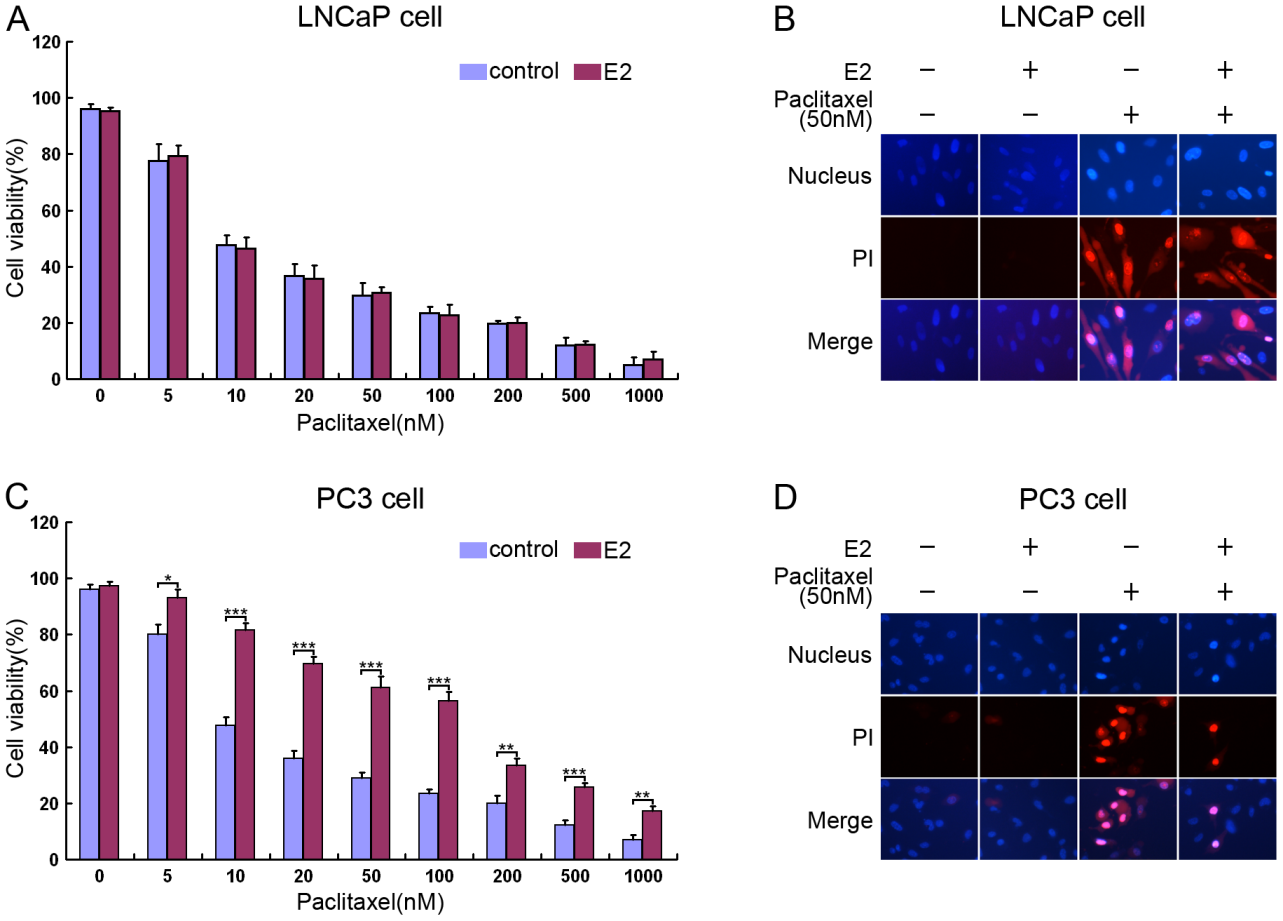
E2 inhibits Paclitaxel induced androgen-independent prostate cancer cell death. (A-D) 100 nM of E2 was added to the media of (A and B) LNCaP and (C and D) PC3 cells for 96h, followed by addition of Paclitaxel at the indicated concentrations for 24h. The cells were stained with Hoechst 33258 (5 µg/ml) to visualize nuclei and propidium iodide (PI) (0.2 µg/ml) to detect membrane damage (B and D). Cell death was quantified by scoring the number of PI positive cells relative to the total number cell nuclei in the same visual field (A and C). The values represent the mean ± S.E. of at least three independent experiments. * denotes p<0.05, ** denotes p<0.01, and *** denotes p<0.001.

To examine whether estrogen is sufficient to regulate the progress of prostate cancer, we first examined the sensitivity of LNCaP cells (androgen-sensitive human prostate adenocarcinoma) and PC3 cells (androgen-independent prostate cancer) for Paclitaxel. We found that Paclitaxel induced the death of both LNCaP and PC3 cells (Fig. 1A and C). E2 was used in this study as a representative of estrogen, because E2 is the most potent estrogen normally found in the circulation. Interestingly, we also found that E2 inhibited Paclitaxel induced PC3 cell death (Fig. 1C and D), yet had no effect on Paclitaxel induced LNCaP cell death (Fig. 1A and B). These results confirm that estrogen inhibits Paclitaxel induced cell death in androgen-independent prostate cancer cells.

### ERα overexpression mediates the estrogen induced Paclitaxel resistance of PC3 cells

Previously, we found that E2 treatment reduces the sensitivity of PC3 cells to Paclitaxel. Estrogens have been reported to suppress proliferation of cultured prostate cancer cells [21]. Two major estrogen receptor types, ERα and ERβ, are expressed in both normal and diseased human prostate, albeit with differing cellular localization [22], [23]. Since these two receptors also display differences in ligand binding, heterodimerization, transactivation and estrogen response element activity, it is likely that assessing and changing their expression may be critical to ultimately determine the effects of estrogen on prostate cancer cells [9].

**Figure 2:**
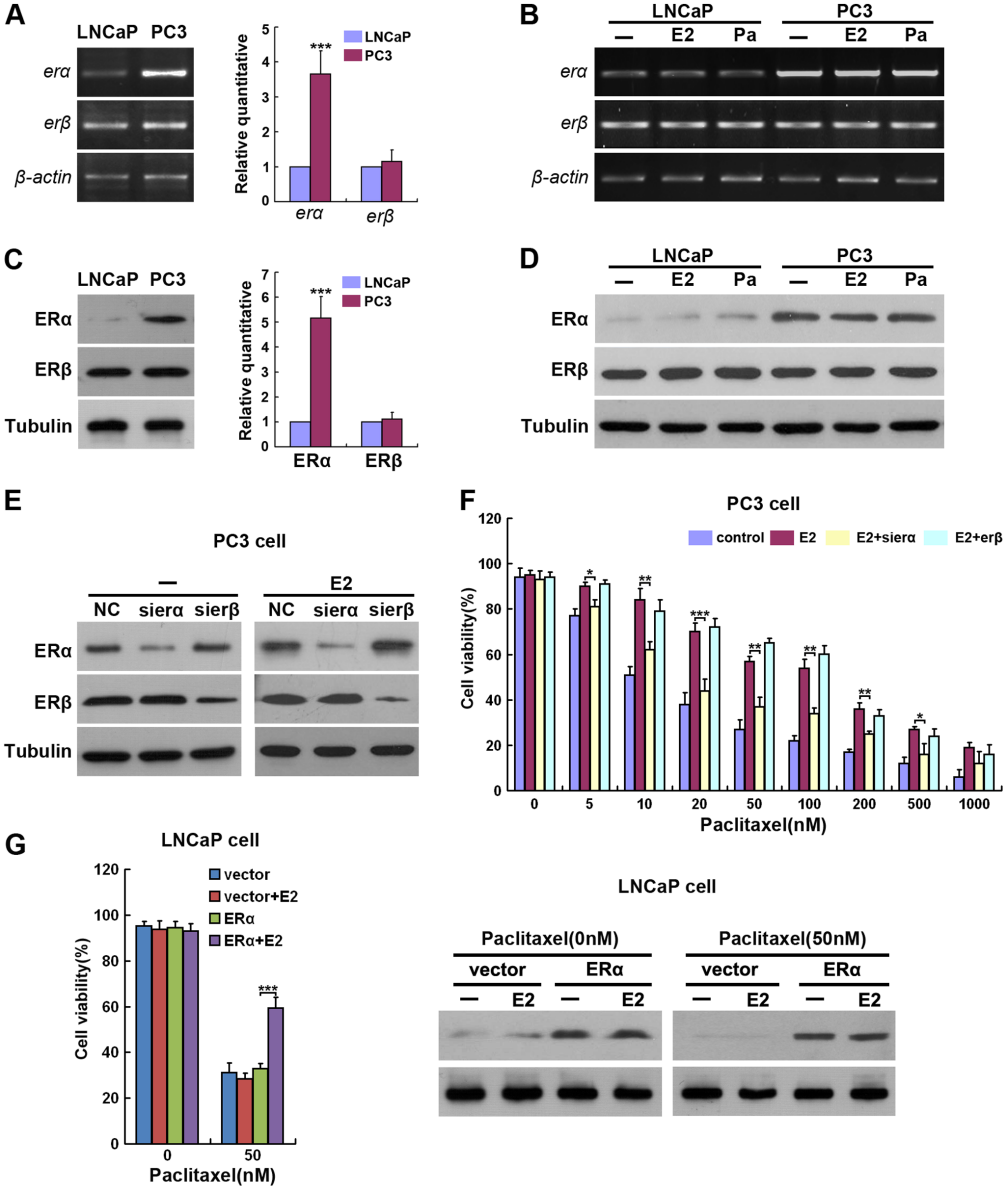
ERα mediates the estrogen induced Paclitaxel resistance of PC3 cells. (A-D) LNCaP and PC3 cells were treated with or without E2 (100 nM) for 96h, Paclitaxel (Pa 50 nM) for 24h. (A and B) Representative and quantification (A right) of mRNA expression levels of *erα* and *erβ*. Total mRNA was extracted and RT-PCR was performed with primers specific to *erα*, *erβ* and *β-actin*. (C and D) Representative and quantification (C right) of protein expression levels of ERα and ERβ. Total protein was extracted and analyzed by Western blot with antibodies specific to ERα, ERβ and Tubulin. (E) Efficacy and specificity of sierα and sierβ knockdown. PC3 cells were treated with (right) or without (left) 100 nM of E2 for 96h, then transfected with the indicated siRNAs or negative control siRNA (NC). After 24h, expression of ERα or ERβ was monitored using Western blotting. (F) PC3 cells were treated with 100nM of E2 for 96h, then transfected with the indicated siRNAs or NC siRNA respectively. Twenty four hours post-transfection, Paclitaxel was added to the media at the indicated concentrations for 24h and the level of cell death was quantified as in Figure 1. (G) LNCaP cells were treated with 100 nM of E2 for 96h, then transfected with vector or ERα expression plasmids. Twenty four hours post-transfection, 50 nM of Paclitaxel was added to the media for 24h and the level of cell death was quantified (left) as in Figure 1 and then the expression of ERα was monitored using Western blotting (right). The values represent the mean ± S.E. of at least three independent experiments. * denotes p<0.05, ** denotes p<0.01, and *** denotes p<0.001.

First, the expression levels of ERα and ERβ in LNCaP and PC3 cells were measured. Consistent with previous studies [23], mRNA and protein expression levels of ERβ were equal in LNCaP and PC3 cells (Fig. 2A and C), whereas ERα mRNA and protein expression levels were significantly higher in PC3 cells than LNCaP cells (Fig. 2A and C). Furthermore, we confirmed that treatment of LNCaP and PC3 cells with E2 or Paclitaxel did not affect the expression levels of ERα and ERβ (Fig. 2B and D). Next, to identify the specific effect of ERα and ERβ in Paclitaxel resistance, siRNAs that target human ERα or ERβ were developed, and their efficacy verified by measuring endogenous ERα or ERβ in PC3 cells following knockdown. Immunoblotting analysis revealed that ERα and ERβ siRNAs specifically abolished the expression of endogenous ERα and ERβ, respectively, in PC3 cells (Fig. 2E), demonstrating high selectivity and efficacy. Interestingly, we found ERα siRNA, but not ERβ siRNA, significantly restored the sensitivity of PC3 cells to Paclitaxel induced death (Fig. 2F). To determine whether ERα is sufficient to induce resistance to Paclitaxel, the effect of ERα on LNCaP cells that had been treated with E2 and Paclitaxel was examined. It was found that overexpression of ERα in LNCaP cells resulted in E2-mediated resistance to Paclitaxel induced cell death (Fig. 2G). Thus, ERα is both necessary and sufficient for E2-mediated Paclitaxel resistance.

### Estrogen activates ERα to suppress PC3 cell proliferation and mediate resistance to Paclitaxel

Evidence has been accumulating that suggests that the expression level of ERα affects the efficacy of chemotherapy. One clinical trial reported that the curative effect of Paclitaxel plus cyclophosphamide adriamycin chemotherapy was higher for ERα negative patients than ERα positive [24]. Furthermore, in breast cancer patients, MaeharaY *et al*. found that ERα negative breast cancer is more sensitive to chemotherapy drugs than ERα positive breast cancer [25]. It is thought that Paclitaxel could induce mitotic phase death in cancer cells, thereby exerting an antitumor effect [2]. Based on these data, we hypothesize that estrogen activates ERα, which suppresses PC3 cell proliferation and thus mediates the cell’s resistance to Paclitaxel.

**Figure 3:**
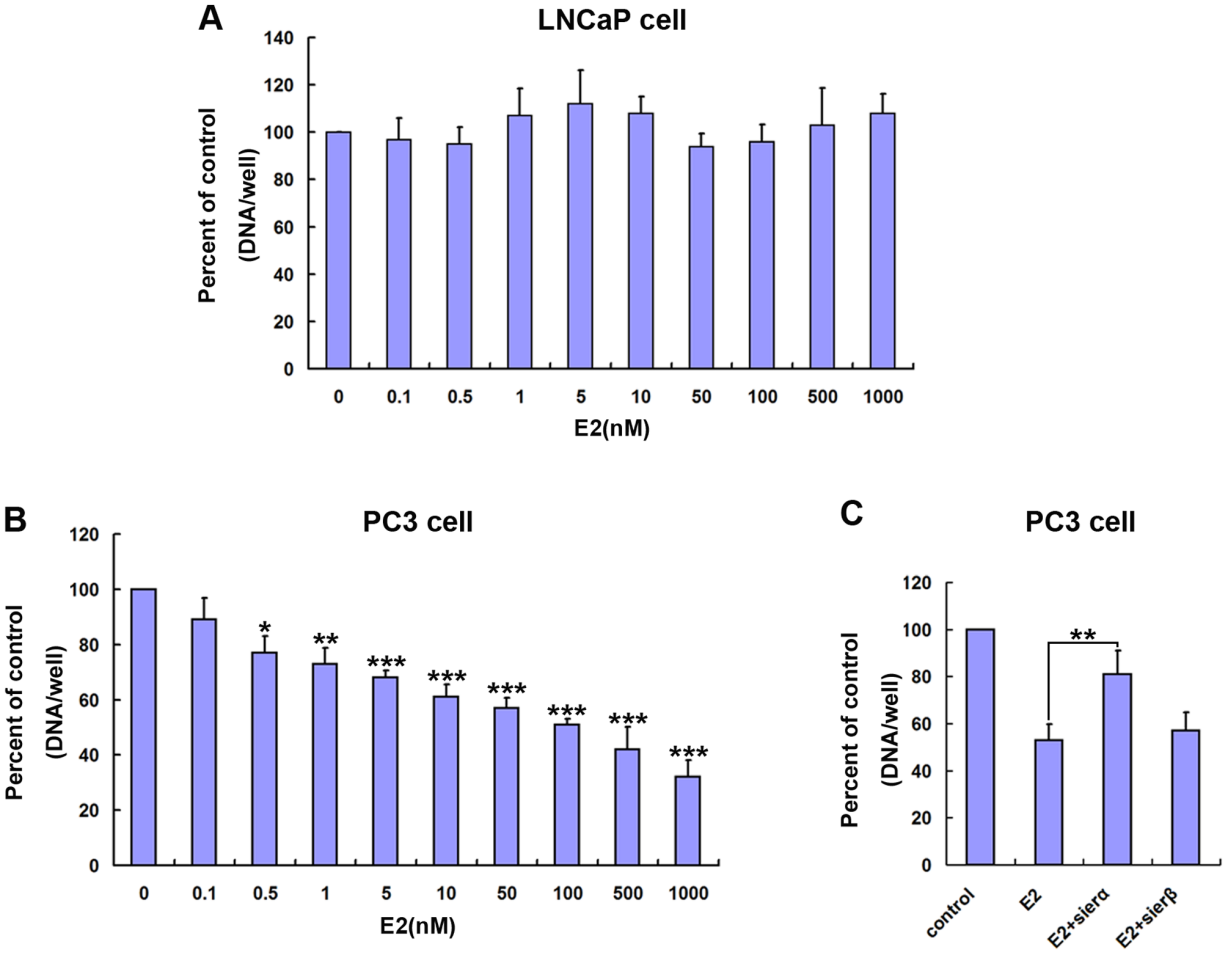
Estrogen activates ERα, suppressing PC3 cell proliferation and mediating its Paclitaxel resistance. E2 was added to the media of (A) LNCaP cells or (B) PC3 cells at the indicated concentrations for 96h, and cell proliferation was quantified. (C) PC3 cells were transfected with the indicated siRNAs or NC siRNA. 24h after transfection, 100 nM E2 was added to the media for 96h as indicated, and cell proliferation was quantified. The values represent the mean ± S.E. of at least three independent experiments. * denotes p<0.05; ** denotes p<0.01; *** denotes p<0.001.

To determine whether estrogen could inhibit the proliferation of prostate cancer cells, we treated LNCaP and PC3 cells with E2. We found that physiological E2 concentrations did not stimulate or inhibit growth of LNCaP (Fig. 3A). However, PC3 cells displayed a significant, E2 dose-dependent inhibition of growth with a maximal effect at 1 mM E2 (32.2% with respect to control) (Fig. 3B). The effect of E2 was also evident after treatment with concentrations higher than 0.5 nM E2 for 96 h (Fig. 3B).

To identify the role of ERα and ERβ in E2 induced PC3 cell suppression, siRNAs targeting human ERα and ERβ were used. We found ERα siRNA, but not ERβ siRNA, significantly inhibited the suppression of PC3 cell proliferation by E2 (Fig. 3C). These results suggest that estrogen suppresses PC3 cell proliferation and mediates Paclitaxel resistance through activation of ERα.

### E2 promotes PHB mitochondrial-nucleus translocation, thus inhibiting cell proliferation

**Figure 4:**
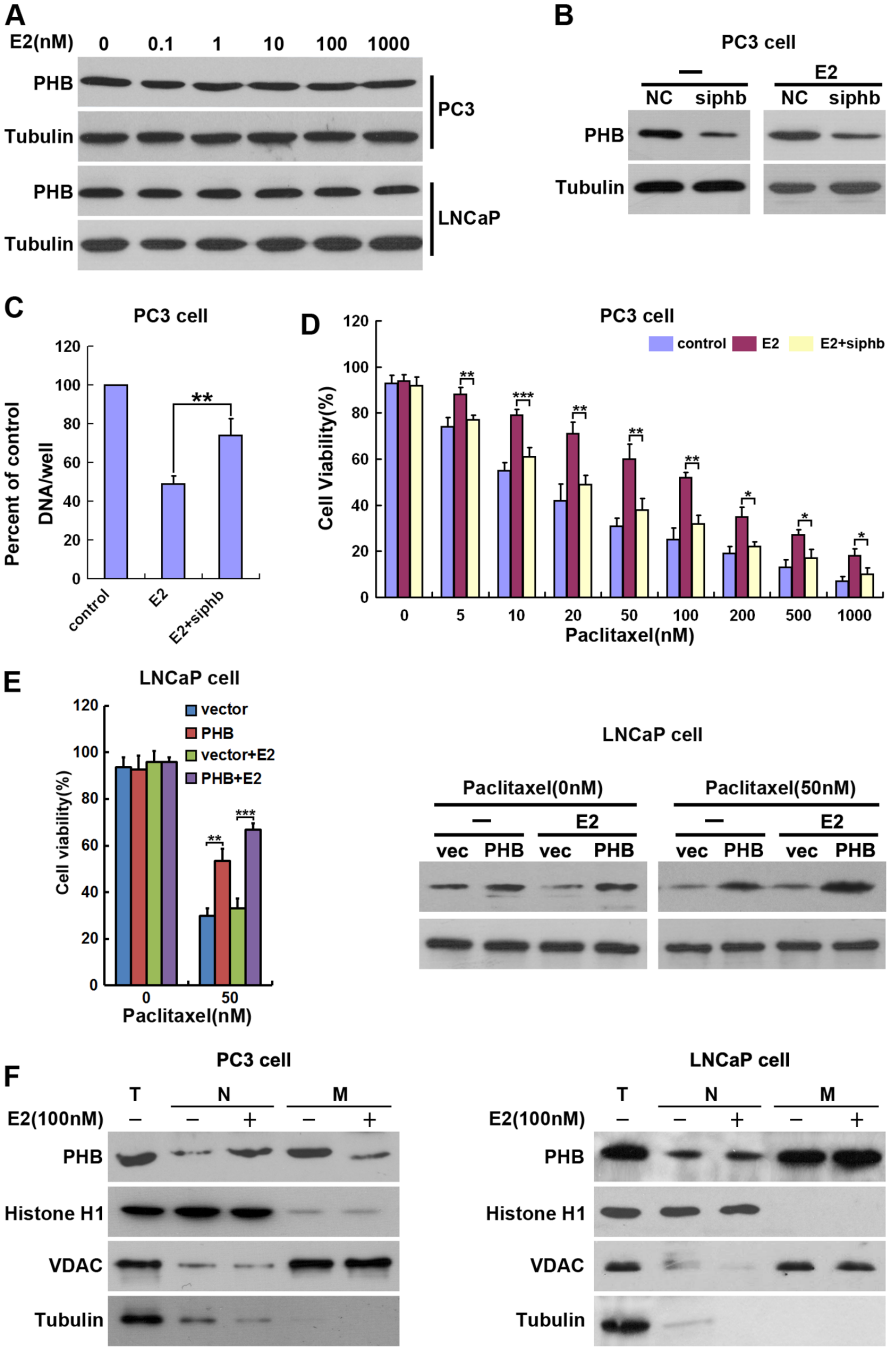
E2 promotes PHB mitochondrial-nuclear translocation, thus inhibiting cell proliferation. (A) E2 was added to the media of LNCaP or PC3 cells at the indicated concentrations for 96h. Total cell protein was extracted and analyzed by Western blot using antibodies specific to PHB and Tubulin. (B) Efficacy and specificity of PHB siRNA is shown. PC3 cells were transfected with PHB siRNA or NC RNA. Twenty four hours post-transfection, 100 nM of E2 was added to the media for 96h and the expression of PHB was analyzed by Western blot. (C) PC3 cells were treated as in B, and the levels of cell proliferation were quantified. (D) 100 nM of E2 was added to the media for 96h, followed by transfection of PC3 cells with PHB siRNA or NC RNA. Twenty four hours post-transfection, Paclitaxel was added to the media at the indicated concentrations for 24h, and the level of cell death was quantified as described in Figure 1. (E) 100 nM of E2 was added to the media for 96h, and then LNCaP cells were transfected with either vector or PHB expression plasmids. Twenty four hours post-transfection, 50 nM of Paclitaxel was added to the media for 24h and the level of cell death was quantified as described in Figure 1. (F) E2 promoted PHB mitochondrial-nucleus translocation. 100 nM of E2 was added to the media for 96h, then PC3 or LNCaP cell mitochondria (M) and nuclei (N) were separated and analyzed by Western blot using PHB, Histone H1 (nucleus marker), VDAC (mitochondrial marker) and Tubulin (cytoplasm marker) antibodies. T (total cell lysates). Results are representative of three independent experiments. The values represent the mean ± S.E. of at least three independent experiments. * denotes p<0.05; ** denotes p<0.01; *** denotes p<0.001.

The androgen receptor is currently the major hormonal target for prostate cancer treatment. However, increasing evidence suggests that estrogen signaling also has an important role in tumor development and progression. Some variants of genes involved in estrogen metabolism, including PHB [26] and estrogen receptors [9], are associated with an increased risk of prostate cancer. Previously, we reported that PHB is an important regulator of transit through the cell cycle [18]. While delineating how estrogen inhibits the proliferation of PC3 cells, we hypothesized that it is through PHB that estrogen mediates its effects. Work by our lab, as well as others, has found that in prostate cancer cells, there is an increased PHB expression in response to stimulation by cholesterol [18]. In this study, we found PHB protein levels remained constant in PC3 and LNCaP cells during E2 treatment (Fig. 4A). Using PHB siRNA, significant knockdown of PHB expression was achieved (Fig. 4B) and this PHB knockdown inhibited the E2 induced suppression of PC3 cell proliferation (Fig. 4C). Furthermore, knockdown of PHB significantly restored the sensitivity of PC3 cells to Paclitaxel induced death (Fig. 4D), suggesting a critical role for PHB in Paclitaxel resistance of PC3 cells.

To determine whether PHB is sufficient to induce Paclitaxel resistance, the effect of PHB on LNCaP cells treated with E2 and Paclitaxel was examined. Forced overexpression of PHB in LNCaP cells could generate E2-mediated Paclitaxel resistance to cell death (Fig. 4E). Thus, PHB itself was sufficient for E2-mediated Paclitaxel resistance.

PHB has been suggested to be localized in the nucleus, to modulate transcriptional activity by interacting with various transcription factors, including nuclear receptors, and to suppress cell proliferation [27], [28], [29]. Also, it has been suggested that PHB may be able to translocate between the mitochondria and the nucleus [30], [31]. However, the mechanisms of PHB function in PC3 prostate cancer cells have not been delineated. Here, we found that the majority of PHB localized in the mitochondrial fraction in both PC3 and LNCaP cells (Fig. 4F). Following E2 treatment, PHB levels were elevated in the nuclear fractions and decreased in the mitochondrial fractions as compared to untreated PC3 cells (Fig. 4F left). However, E2 did not affect the localization of PHB in LNCaP cells (Fig. 4F right). These results indicate that E2 promotes PHB mitochondrial-nuclear translocation, thus inhibiting cell proliferation.

### ERα directly mediates PHB mitochondrial-nuclear shuttling

Previous studies have shown that PHB is mainly localized and functions in the mitochondria, and that mitochondrial PHB translocates to the nucleus in the presence of ERα [30], [31]. However, it remains unclear how PHB is delivered to the nucleus in prostate cancer cells. We hypothesized that the nuclear redistribution of PHB is driven by ERα.

**Figure 5:**
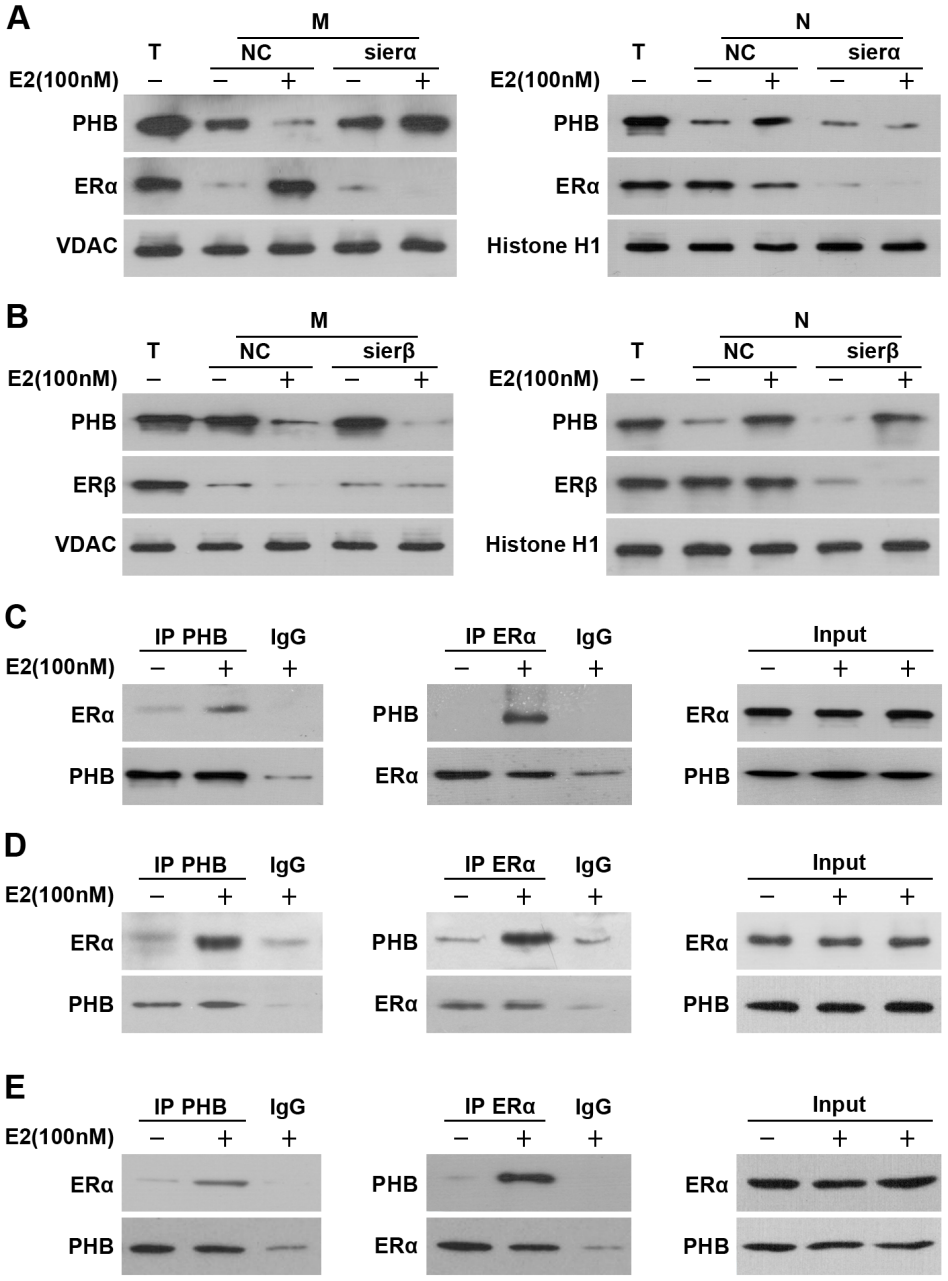
ERα directly mediated PHB mitochondrial-nuclear shuttling. (A and B) ERα mediated PHB mitochondrial-nucleus translocation. PC3 cells were transfected with siRNAs specific to (A) erα or (B) erβ, or NC RNA. Twenty four hours post-transfection, 100 nM of E2 was added to the media for 96h. The PC3 cell mitochondria (M) and nuclei (N) were separated and analyzed by Western blot using PHB, ERα Histone H1 (nucleus marker), VDAC (mitochondrial marker) and Tubulin (cytoplasm marker) antibodies. T (total cell lysates). (C) ERα directly associates with PHB. 100 nM of E2 was added to the media for 96h, then PC3 cell lysates were immunoprecipitated (IP) using PHB antibody and analyzed by Western blot (WB) using the indicated antibodies (left panel). PC3 lysates were immunoprecipitated with ERα antibody, and PHB and ERα levels were analyzed by Western blot (middle panel). Equal amounts of total input PHB and ERα (Input) were used for immunoprecipitations for each condition (right). (D) ERα directly associates with PHB in mitochondria. PC3 cells were treated as in C, then the PC3 cell mitochondria were separated and immunoprecipitated as in C. (E) ERα directly associates with PHB in nucleus. PC3 cells were treated as in C, then PC3 cell nuclei were separated and immunoprecipitated as in C. Results are representative of three independent experiments.

To investigate whether ERα mediated the mitochondrial-nuclear translocation of PHB, PC3 cells were treated with or without E2, as well as with ERα or ERβ siRNA. PHB localization was qualitatively assessed to determine if treatment induced translocation to the nucleus, rather than remaining in the mitochondria. We found that E2 induced the translocation of PHB from the mitochondria to the nucleus, and that this translocation was inhibited by ERα siRNA (Fig. 5A). By contrast, ERβ siRNA did not affect the translocation of PHB (Fig. 5B), suggesting that PHB mitochondrial-nuclear shuttling occurs in an ERα-dependent, but ERβ-independent manner.

To further confirm whether ERα could directly mediate the translocation of PHB, we analyzed the immunoprecipitated pellet of endogenous PHB for the presence of estrogen receptors. In PC3 cells, immunoblot analysis of immunoprecipitated PHB detected the presence of ERα (Fig. 5C) but no ERβ (data not shown). Also, PHB was present following a reciprocal immunoprecipitate using the antibody against ERα (Fig. 5C). Furthermore, we found that PHB interacted with ERα in both the mitochondrial and nuclear fractions upon E2 treatment (Figure 5D and 5E). In LNCaP cells, no interaction was detected between PHB and ERα (because the expression level of ERα in LNCaP cell is very low) or ERβ (data not shown). These results indicate that PHB could physically interact with ERα, and ERα could directly mediate the PHB mitochondrial-nuclear shuttling.

### PHB acts downstream of ERα to mediate resistance to Paclitaxel

**Figure 6:**
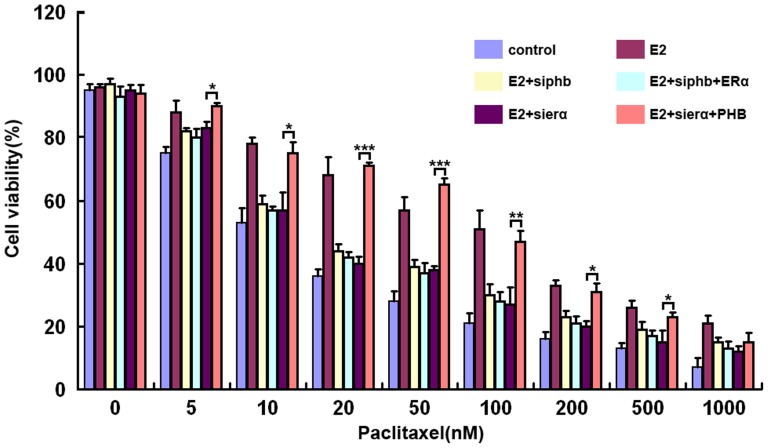
PHB acts downstream of ERα to mediate resistance to Paclitaxel. 100α or PHB expression plasmids, or NC RNA. Twenty four hours post-transfection, Paclitaxel was added to the media at the indicated concentrations for 24h, and the level of cell death was quantified as described in Figure 1. The values represent the mean ± S.E. of at least three independent experiments. * denotes p<0.05; ** denotes p<0.01; *** denotes p<0.001.

Based on the importance of ERα and PHB in E2 induced Paclitaxel resistance of PC3 cells, we hypothesized that PHB might mediate the resistance induced by estrogen and ERα. ERα and PHB were manipulated using a combination of knockdown and overexpression approaches to investigate their functional relationship. The ERα siRNAs, known to abolish ERα protein levels in PC3 cells, significantly blocked E2 induced Paclitaxel resistance of PC3 cells (Fig. 2D). Similarly, the knockdown of endogenous PHB also markedly inhibited E2 induced Paclitaxel resistance of PC3 cells (Fig. 4D and Fig. 6) and overexpression of ERα failed to overcome the effect of PHB siRNAs (Fig. 6). Furthermore, overexpression of PHB, combined with transfection of ERα siRNAs, reversed the phenotype normally seen following suppression of ERα (Fig. 6). These findings demonstrate that PHB lies downstream of the E2/ERα-dependent Paclitaxel resistance signaling cascade.

## Discussion

Patients with castration-resistant prostate cancer are at a high risk of death. Treatment of these cancers includes second-line hormones, novel agents and chemotherapy with taxanes, such as Paclitaxel and docetaxel. Paclitaxel is a widely used, effective agent in the treatment of a variety of human cancers. As with many other chemotherapeutic agents, however, resistance to Paclitaxel remains a limiting factor for its clinical efficacy. Despite this limitation, Paclitaxel remains at the frontline of cancer therapy and has stimulated a concerted effort to understand the molecular mechanisms of Paclitaxel resistance [32]. In the current study, we demonstrate Paclitaxel resistance is associated with estrogen treatment in androgen-independent PC3 cells, but not in androgen-sensitive LNCaP cells. We also show that estrogen activates ERα to suppress PC3 cell proliferation and mediate Paclitaxel resistance. Moreover, ERα directly mediates the mitochondrial-nuclear shuttling of PHB and inhibits cell proliferation. Combined, these findings demonstrate that estrogen activates ERα and promotes PHB mitochondrial-nuclear translocation, leading to resistance of androgen-independent prostate cancer cells to Paclitaxel.

Experimental androgen-deprivation therapy (ADT) for prostate cancer in the form of estrogen treatment was first reported nearly 70 years ago [33]. The results of these initial studies were translated to the clinic, where ADT was shown to slow the inexorable progression of prostate cancer [34]. Unfortunately, however, the development of so-called castrate-resistant prostate cancer limits the effects of ADT [35]. In fact, it is controversial as to whether estrogen has inhibitory effects on prostate cancer. On one hand, estrogen may be effective as a second line hormonal treatment for patients with androgen-independent prostate cancer and may improve patient survival [36], [37]. On the other hand, increasing evidence shows that estrogen signaling has a major role in prostate cancer development and progression [6], [7], [8]. At present, there is no clear mechanism that explains these two disparate conclusions. We demonstrate here that estrogen, through repression of cell proliferation, induces Paclitaxel resistance in androgen-independent PC3 cells, but not androgen-sensitive LNCaP cells, in an ERα/PHB-dependent pathway.

Several studies have shown that repression of PHB in ovarian cancer cells and lung carcinoma cells improves their sensitivity to staurosporine and Paclitaxel [17], [32]. Here, we show that PHB knockdown restored Paclitaxel sensitivity to resistant PC3 cells. This restored sensitivity to Paclitaxel following PHB knockdown results from increased proliferation of PC3 cells. Interestingly, we observed a recovery in Paclitaxel sensitivity after repression of PHB in estrogen induced Paclitaxel resistant cells, suggesting that PHB may sufficient for the onset and maintenance of estrogen induced Paclitaxel resistance. Together, our results suggest that PHB reduction can improve Paclitaxel sensitivity in androgen-independent and taxane-resistant prostate cancer cells.

Our data establishes for the first time that estrogen induces taxane-resistance of androgen-independent prostate cancer cells in an ERα/PHB-dependent mechanism. Of particular interest is how this mechanism relates to ADT. In cases where castrate-resistant prostate cancer limits the effects of ADT, estrogen could be used as a hormonal treatment for prostate cancer, while simultaneously repressing the activity of PHB to avoid the induction of the drug resistant effect of estrogen. This method may recover the efficacy of taxane in the clinic. Furthermore, alterations in PHB levels or interference with PHB localization may also result in increased sensitivity of other types of tumors to taxane treatment.
